# Measurement of Strain and Vibration, at Ambient Conditions, on a Dynamically Pressurised Aircraft Fuel Pump Using Optical Fibre Sensors

**DOI:** 10.3390/s25206407

**Published:** 2025-10-17

**Authors:** Edmond Chehura, Stephen W. James, Jarryd Braithwaite, James H. Barrington, Stephen Staines, Andrew Keil, Martin Yates, Nicholas John Lawson, Ralph P. Tatam

**Affiliations:** 1 Engineering Photonics, Cranfield University, Bedford MK43 0AL, UKj.h.barrington@cranfield.ac.uk (J.H.B.);; 2 Technical Support Services, Cranfield University, Bedford MK43 0AL, UK; 3 Innovation Programmes, Saab UK, 2nd Floor, Kinnaird House, 1 Pall Mall East, London SW1Y 5AU, UK; 4 Rolls-Royce plc, The Derwent Building, 5000 Solihull Parkway, Birmingham Business Park, Birmingham B37 7YP, UK; 5 National Flying Laboratory Centre, Cranfield University, Bedford MK43 0AL, UK; nicholas.lawson@sydney.edu.au; 6 Faculty of Engineering, School of Aerospace, Mechanical & Mechatronic Engineering, The University of Sydney, Sydney, NSW 2006, Australia

**Keywords:** fibre Bragg grating sensor, fibre optic sensor, strain, vibration, temperature, fuel pump, strain gauge, thermocouple, finite element model, dynamic pressure, static pressure

## Abstract

Ever-increasing demands to improve fuel burn efficiency of aero gas turbines lead to rises in fuel system pressures and temperatures, posing challenges for the structural integrity of the pump housing and creating internal deflections that can adversely affect volumetric efficiency. Non-invasive strain and vibration measurements could allow transient effects to be quantified and considered during the design process, leading to more robust fuel pumps. Fuel pumps used on a high bypass turbofan engine were instrumented with optical fibre Bragg grating (FBG) sensors, strain gauges and thermocouples. A hydraulic hand pump was used to facilitate measurements under static conditions, while dynamic measurements were performed on a dedicated fuel pump test rig. The experimental data were compared with the outputs from a finite element (FE) model and, in general, good agreement was observed. Where differences were observed, it was concluded that they arose from the sensitivity of the model to the selection of nodes that best matched the sensor location. Strain and vibration measurements were performed over the frequency range of 0 to 2.5 kHz and demonstrated the ability of surface-mounted FBGs to characterise vibrations originating within the internal sub-components of the pump, offering potential for condition monitoring.

## 1. Introduction

Future civil aerospace technology advancement and improvement will be based increasingly on the use of real-time aircraft and engine data to predict performance, adapt control in-flight, and manage maintenance, with sensors being the cornerstone for this approach. However, the foremost challenges to the implementation of any kind of sensor in turbomachinery applications are the high temperatures, the complex shape of components, the restricted space, and the difficulty in accessing the areas of interest, while the environment itself is harsh, noisy and flammable [1]. Therefore, the ideal sensors should be relatively small in size, be lightweight, be non-intrusive so as not to compromise the structural integrity, be non-corrosive and inert/non-reactive, and be immune to electromagnetic interference.

The potential for the use of optical fibre-based instrumentation and sensors in turbomachinery has long been recognised [2,3,4,5]. In the 1980s, fibrescopes (imaging optical fibre bundles acting as flexible endoscopes) were used to inspect the gas path of an engine without removal and disassembly of the components [6]. The same paper discussed the potential of fibre optic implants to allow continuous monitoring of the health of the gas path. Since then, optical fibre sensors (OFS) have been used or proposed for the measurement of parameters such as flow velocity [7,8], tip timing and clearance [9,10], temperature [11,12,13], pressure [14,15], strain [16,17] and vibration [18].

The key benefits of OFS include the flexibility of deployment in intricate, difficult-to-access locations and the ability to keep the electronic and optoelectronic components away from the harsh measurement environment. Intrinsic sensors, in which the optical fibre itself is the measurement medium, involve the interaction of the local environment with the optical fibre to modulate the properties of the light propagating within the fibre, such as phase, polarisation, amplitude, and wavelength [19]. Here, the drivers of interest in their use are their lightweight, small dimensions, flexibility, immunity to electromagnetic interference, and the potential for distributed or multiplexed sensing of parameters such as temperature, strain, vibration and pressure.

The initial progress on the implementation of optical sensing technologies in favour of conventional sensing was slow, in part due to some of the limitations of the then-available electro-optic and fibre optic components. Over the intervening period, OFS technology matured, with a range of new measurement approaches developed, and robust and cost-effective interrogation systems made possible by adopting components developed and mass-produced for the telecommunications industry. Companies are offering systems for the measurement of a range of physical and chemical parameters, including strain, temperature, pressure, vibration, shape, acceleration, refractive index, humidity, and liquid level [20,21].

Despite the increasing commercialisation of intrinsic OFS technology and its widespread deployment in the oil and gas industry [22,23] for the monitoring of the integrity of pipelines [24], its use in wind energy facilities [25] for monitoring rotor blade loading [26], and its adoption by the security and civil engineering sectors for structural health monitoring of bridges, dams, and tunnels [27], the available literature suggests its application in aerospace seems limited to demonstrations of capability [28,29], rather than routine utilisation. This shows that there is still research to be undertaken in order to fulfill the aerospace requirements, given that it is a very conservative industry.

In this paper, we report on the experimental work undertaken on the real-time monitoring of the performance of an aircraft fuel pump using arrays of surface-adhered optical fibre sensors. A Rolls-Royce fuel pump of a type used on modern high-bypass turbofan engines was instrumented with a total of 24 wavelength division multiplexed optical fibre Bragg grating (FBG) sensors, which were spatially distributed over the intricate and complex surface of the fuel pump and deployed for the measurement of orthogonal strain components and vibration during flight-simulating experimental conditions. To aid comparative measurements, T-strain gauge rosettes were integrated onto the fuel pump to determine the orthogonal components of strain at spatial locations in the vicinity of the FBG sensors, although the positions at which they could be located were restricted due to the complex shape of the pump. Thermocouples were also attached to the pump at various locations, with the measurements used to compensate for the temperature sensitivity of the FBG sensors. Data were also acquired from a variety of fuel pressure, fuel flow rate, speed and temperature sensors that were built into the dynamic fuel-pressure testing rig used for flight simulations. The measurements from this experimental campaign were compared with the FBG and strain gauge measurements obtained from a separate experiment performed under static pressure at ambient temperatures in the laboratory, with the fuel pump pressurised by water from a hand pump. Further comparisons were made with the output from a finite element (FE) model implemented for the static pressure characterisation. This work demonstrates for the first time that dynamic information on internal sub-components can be obtained by using surface-adhered OFS for condition monitoring of a turbomachinery component. This is a significant observation, as the direct measurement of interior components would require the disassembly of the subsystems of the pump for sensor installation, which would be impractical, time-consuming, and could cause unwanted intrusion to the normal operation of the pump [30].

## 2. Sensors and Instrumentation

### 2.1. Fuel Pump Object

**Figure 1:**
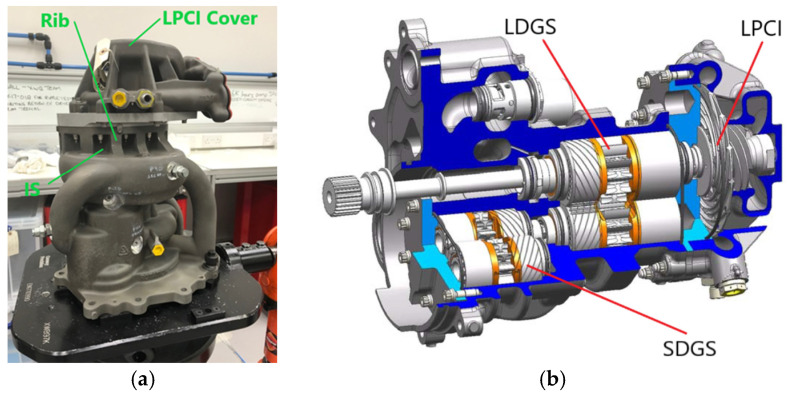
The Rolls-Royce fuel pump: (**a**) pump body, and (**b**) cutaway view of the inside of the pump showing the gear and impeller systems. IS—inset section, LDGS—large displacement gear system, SDGS—small displacement gear system, LPCI—low pressure centrifugal impeller. Figure 1b, with copyright permission from Elsevier, is reprinted from [31].

Figure 1 shows the Rolls-Royce fuel pump body and a cutaway view that shows the gear and impeller systems [31]. An external gearbox, not shown in Figure 1, drives the large displacement gear stage (LDGS), which in turn transfers the drive simultaneously to a small displacement gear stage (SDGS) and to a low-pressure centrifugal impeller (LPCI). Both the LDGS and the SDGS have 14 teeth, while the LPCI has 14 fins. The fuel pump serves to regulate the fuel pressure, to supply fuel to the aero-engine, and to provide cooling for the oil system [31]. The pump body in Figure 1a is made out of aluminium and it fits in an envelope of dimensions 370 mm × 370 mm × 470 mm.

### 2.2. Sensor Configuration

**Figure 2:**
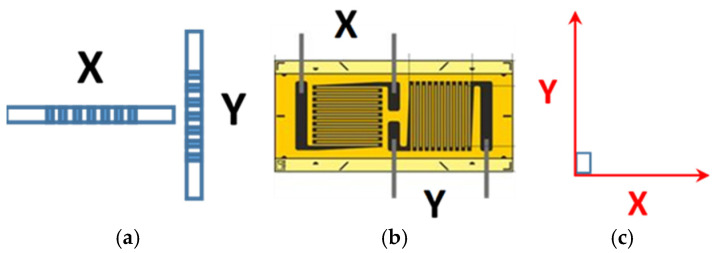
The sensor configuration that was employed on the main body of the fuel pump such that orthogonal components X (hoop direction) and Y (longitudinal direction) were measured by (**a**) FBG sensors and (**b**) T-strain gauge rosettes, as described by the coordinate system in (**c**).

In both of the experimental campaigns described in this paper, the FBG sensors were configured such that, at each spatial location on the main body of the fuel pump, two FBGs were oriented orthogonally with respect to each other to measure the hoop and longitudinal strains, hereafter referred to as X and Y, respectively (Figure 2a), considering the pump to be broadly cylindrical in shape. The FBGs were not pre-strained before being attached to the fuel pump body for both experimental campaigns as previous work has shown that they can measure compressive strain [32,33,34]. T-strain gauge rosettes, also configured to measure the local X and Y strain components, were attached to the main body of the fuel pump, such that they were spatially located in the vicinity of the FBG sensors, as illustrated in Figure 2b. Figure 2c denotes the coordinate system used.

### 2.3. Fibre Bragg Grating Sensors (FBGs)

An FBG comprises a periodic modulation of the refractive index of the core of the optical fibre [35], induced typically by exposing the fibre to a spatially modulated intensity profile from a UV or femtosecond pulsed laser. The FBG acts as a wavelength selective mirror that reflects a narrow wavelength band (typically 0.1 nm wide) back along the optical fibre; all other wavelengths are transmitted by the FBG. In this work, the FBG sensors, each 3 mm long, were fabricated in-house by exposing the optical fibre to the output from a frequency-quadrupled flashlamp-pumped Nd: YAG laser, operating at 266 nm, through a phase mask [35]. The fibres were, prior to FBG fabrication, soaked in hydrogen for 2 weeks at room temperature and with the gas pressure maintained at 100 Bar, to photosensitise the fibre core [36].

#### Principles of FBG Sensors

The reflected Bragg wavelength, *λ_B_*, is dependent upon the period of the grating, Λ, and the effective refractive index of the propagating mode, *n_eff_*, according to Equation (1).

(1) $$\lambda_{B}={2n}_{eff}\Lambda$$

**Figure 3:**
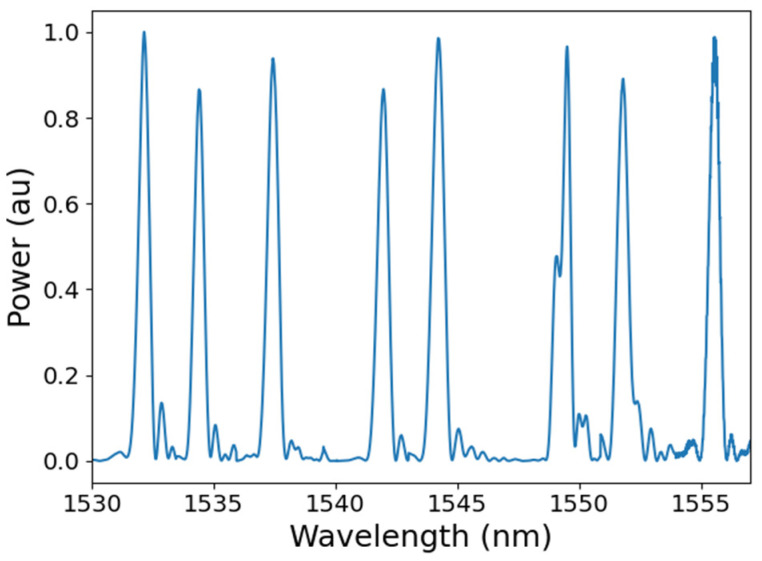
The reflection spectrum from an optical fibre containing an array of eight FBGs that were fabricated at different wavelengths and spatially distributed along the fibre length, obtained using a SmartScan FBG interrogator.

Measurands that interact with the FBG in a way that changes the period and/or the refractive index of the fibre will cause a concomitant change in the reflected Bragg wavelength. The primary parameters to which the FBG is sensitive are strain and temperature. It follows from Equation (1) that multiple FBGs, with different periods and thus different Bragg wavelengths, can be fabricated within a single optical fibre with arbitrary physical separation, exploiting wavelength-division multiplexing approaches [37] to facilitate measurements at multiple spatial locations. Figure 3 shows the reflection spectrum obtained from a wavelength division multiplexed array of FBGs fabricated in telecommunications optical fibre (SMF28). Equation (2) represents the Bragg wavelength shift, Δ*λ_Β_*, in response to changes in longitudinal strain, Δ*ε*, and temperature, Δ*T* [35].

(2) $$\Delta \lambda_{B}=K_{\varepsilon }\cdot \Delta \varepsilon +K_{T}\cdot \Delta T$$

In Equation (2), *K_ε_* and *K_T_* are the strain and temperature responsivities of the FBG, respectively. For an FBG fabricated in the SMF28 optical fibre used in the fuel pump dynamic tests, the experimentally calibrated strain and temperature responsivities are 1.2 pm/με and 11 pm/°C, respectively [38]. The temperature responsivity, *K_T_*, of an FBG in a freely suspended fibre is the sum of the thermal expansion (*α_f_*) and thermo-optic (*ξ_f_*) coefficients of the fibre, with values of ~0.55 × 10^−6^/°C and ~7.3 × 10^−6^/°C, respectively, for SMF28 fibre at a wavelength of 1550 nm, which has been shown to be valid for a limited temperature range of approximately 20 °C to 80 °C [39]. Equation (2) can be expressed as Equation (3) for an FBG bonded to, or embedded into, a host material that has thermal expansion coefficient of *α_h_*. Equation (3) indicates that the thermal expansion and thermo-optic coefficients of the fibre are functions of temperature, but both are approximately constant over the temperature range used in this work [39].

(3) $$\Delta \lambda_{B}=K_{\varepsilon }\cdot \Delta \varepsilon +\left[\alpha_{f}\left(T\right)+\xi_{f}\left(T\right)+\alpha_{h}\right]\cdot \Delta T$$

In experiments where either the influence of temperature is considered sufficiently small to be ignored, or in the measurement of dynamic strain where the temperature is slowly varying at a very different timescale, the second term in Equation (3) is often dropped. The strain that is calculated without employing temperature compensation is referred to as ‘raw’ strain in this paper. The mechanically applied strain can be determined by negating the wavelength shift of the FBG that is caused by the thermal load. Two techniques are generally used for this. The first utilises a reference FBG, located in close proximity to the sensing FBG, which is configured such that it senses only the local temperature by ensuring that it is in thermal contact with the substrate while it is isolated from strain [40]. The advantages of this approach are that it does not require the use of a thermocouple and that all the strain and temperature coefficients are determined from the calibration of a reference FBG. This technique increases the number of FBGs to be interrogated by up to a factor of two, which can compromise the number of measurement locations, as typical commercially available FBG interrogators offer the ability to interrogate simultaneously up to 4 FBG arrays, with up to 20 FBGs per array.

The second commonly employed temperature compensation technique requires the placement of a thermocouple probe close to each sensing FBG. This approach was adopted in this work due to the limitations on the number of optical fibres that could be connected to the interrogator. The term in the parenthesis of Equation (3) represents the thermal strain, where the change in temperature, Δ*T*, can be obtained directly from a thermocouple probe, which when subtracted from the total measured strain, gives the mechanical strain described by Equation (4).

(4) $${\Delta \varepsilon }_{mech}=\frac{1}{K_{\varepsilon }}\left\{\Delta \lambda_{B}-\left[\alpha_{f}\left(T\right)+\xi_{f}\left(T\right)+\alpha_{h}\right]\cdot \Delta T\right\}$$

The coefficients, *ξ_f_*(*T*) and *α_f_*(*T*), are considered constant as the experiments were performed at ambient temperature, with a maximum temperature excursion of approximately 10 °C. However, where greater temperature excursions occur, these coefficients would require experimental characterisation over the temperature range experienced during the experiments in order to obtain the calibration function that will enable effective temperature compensation [41]. As the experiments reported in this work were carried out under ambient temperature conditions, the calibrations were performed off-line in the laboratory.

Δ*ε_raw_* is the measured strain before temperature compensation, while the temperature-compensated strain, Δ*ε_mech_*, is obtained using Equation (5), which is simplified from Equation (4). The effective temperature responsivity of the FBG, Γ*_c_* = 24.5 με/°C, is estimated from α*_h_* = 21.6 × 10^−6^/°C [42] for aluminium (the material of the fuel pump casing) and from *K_T_* = 11 pm/°C calibrated in the laboratory for a freely suspended FBG, while Δ*T* is a measurement from the thermocouple probe.

(5) $$\Delta \varepsilon_{mech}=\Delta \varepsilon_{raw}-\Gamma_{c}\cdot \Delta T$$

All FBG sensors described in this paper were interrogated using a SmartScan (Smart Fibres Ltd. Bracknell, UK) instrument [43]. The instrument is capable of interrogating four channels with a total capacity of 64 FBG sensors at a maximum scanning speed of 2.5 kHz, with a stated strain resolution of 1 με. Figure 3 is an example of the spectrum obtained from an FBG array containing 8 FBG sensors fabricated with different centre wavelengths and spatially distributed along the fibre length.

### 2.4. Instrumented Fuel Pump: Static Test

The purpose of the static test (ST) was to provide a platform, prior to installation onto the harsh environment of the flight simulation conditions of the test rig, to establish the practicality of integrating the fibre sensors onto the fuel pump. Factors that were considered include (i) the bonding of the sensors and the routing of the fibre over the complex contours of the pump, ascertaining sensor survivability and signal integrity, (ii) means for the protection of the fibres to allow for the handling of the fuel pump during installation onto the test rig, (iii) to obtain an understanding of the performance of the optical fibre sensors mounted on the fuel pump under stable and controllable laboratory conditions, and (iv) to provide experimental data to compare with results from the subsequent dynamic pressure testing, and to verify FE models and to thus facilitate improvements to the FE models to make them suitable for generic use in this turbomachinery application. The measurement of strain distribution in ST was also necessary to ascertain the positions on the fuel pump to install sensors for the dynamic test (DT), for which there is no model.

Four optical fibres were prepared such that each fibre contained an array of eight spatially distributed FBG sensors. Each 3 mm long FBG was located centrally within a 6 mm section of fibre that had been stripped of its polyacrylate coating. The entire uncoated fibre length for each sensor was bonded to the fuel pump using a thin layer of allyl cyanoacrylate adhesive (Permabond C920), which has an operating temperature range of −30 to +250 °C. Two resistive foil strain gauges (RFSGs), configured orthogonally, were bonded to the surface of the fuel pump using Permabond C920. Two thermocouple probes (K type) were also attached to the pump, one near the bottom and the other near the top of the fuel pump, using aluminium tape [44].

### 2.5. Instrumented Fuel Pump: Dynamic Test

**Figure 4:**
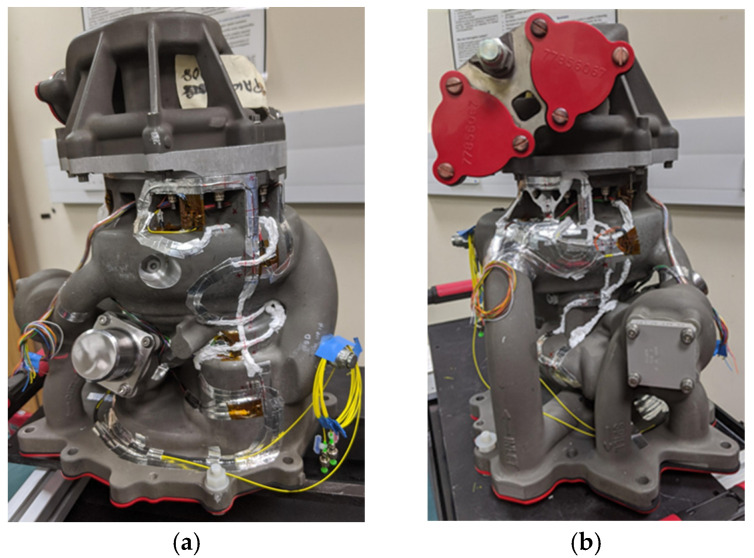
(**a**) Side 1 and (**b**) side 2 of the Rolls-Royce fuel pump, following installation and protection of the FBG sensors, strain gauges and thermocouples for DT. The yellow cables are optical fibre pigtails from the installed FBGs, while the multi-coloured cables are from the installed strain gauges.

For the dynamic test (DT), a total of 24 FBG sensors were fabricated in four optical fibres. Each fibre contained an array of either five or seven wavelength division multiplexed FBGs, each of gauge length 3 mm. The sensor arrays were attached to the fuel pump according to the spatial locations and orientations which were informed by the earlier ST experiments (Section 2.4). Figure 4 shows the fuel pump following the installation of the sensors and their protection, while the sensor locations are described in detail in Section 5.2 and Section 5.3. For practical reasons, the total number of FBG sensors for DT was reduced to 24 from the 32 that were used for the ST. When instrumenting the pump used for DT, refinements had to be made to the positions of the FBG sensors and routing of the fibre, giving consideration to the process of installation of the pump on the thermal test rig, which involved strapping the pump to a hoist. As a result, some of the original sensor locations were no longer viable. The FBG sensors were bonded to the fuel pump using Permabond C920, as described in Section 2.4. The same adhesive was used to bond eight T-strain gauge rosettes, each consisting of two 3 mm long orthogonally oriented resistive foil strain gauges, onto the fuel pump. The total number of strain gauges for DT was increased to 16 (that is, eight T-strain gauge rosettes) to provide independent strain measurements at all locations on the uncurved parts of the pump where it is possible to bond the strain gauges. Only two strain gauges were used for ST as the FE model was used as the primary comparator. Their use also provided an understanding of the process of their bonding to the surface of the pump prior to undertaking DT, where they would act as the primary comparator. Thirteen thermocouple probes were attached to the fuel pump using aluminium tape. The DT measurements were taken within a facility where the ambient temperature was not controlled, and it was also anticipated that there would be temperature changes during the experiment arising from the work done on the fuel by the pump and recirculating system. Given the complexity of the path for the fuel within the pump, this was likely to result in a non-uniform surface temperature distribution. Thus, it was decided to use 13 thermocouples for DT rather than the two thermocouples used for ST, where the pump was uniformly pressurised, with no fluid flow, within a stable laboratory environment. Silicone sealant, together with aluminium tape, was used to cover the lengths of optical fibre separating adjacent FBG sensors to provide mechanical protection.

**Figure 5:**
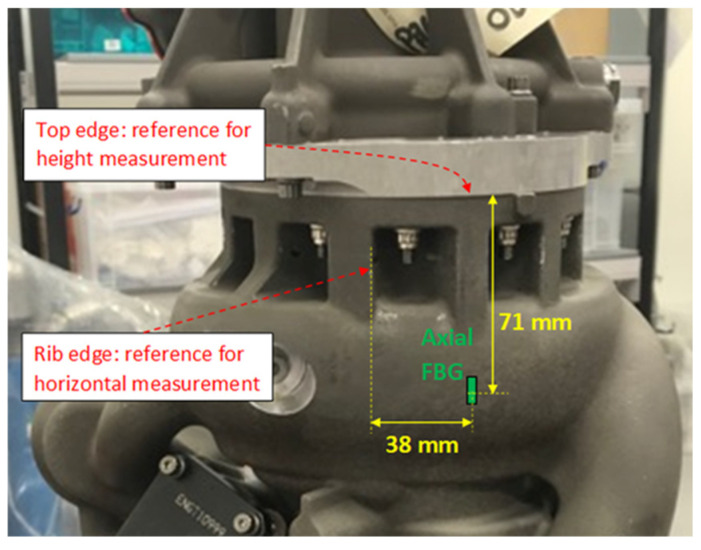
An example of the means by which the position coordinates of the sensors were determined relative to pump features, in this case for the vertically bonded FBG.

To allow comparison of the data from the FBG sensors with the output from an FE model of the pump for ST only, the physical coordinates of the FBG sensors and the strain gauges were determined relative to known reference features on the fuel pump. Figure 5 shows an example of the mapping of the physical locations for one FBG sensor. As the surface of the fuel pump has a complex shape, a flexible tailor’s tape measure was used to determine the coordinates with an estimated error of ±1 mm.

## 3. Fuel Pump Pressure Testing Facilities

### 3.1. ST Setup

**Figure 6:**
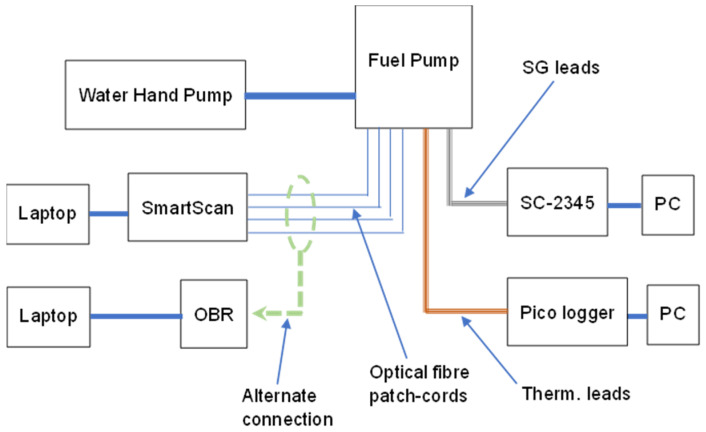
Experimental setup for the ST of the fuel pump instrumented with FBG, RFSG sensors, and thermocouple probes. SG—strain gauge, OBR—optical backscatter reflectometer, SC-2345—strain gauge signal conditioner, PC—personal computer, Therm.—thermocouple.

Figure 6 shows the experimental setup used for the ST, where a hydraulic hand water pump with a pressure capacity of 100 bar was used to pressurise the instrumented fuel pump by flood-filling it with water. The acquisition of the temperature data was performed using a temperature data logger (Pico logger, TC 08) that can acquire eight temperature channels simultaneously. All 32 FBG sensors were interrogated and recorded simultaneously using the SmartScan FBG interrogator, while an Optical Backscatter Reflectometer (Luna, OBR 4400), a distributed measurement system, was used as a diagnostic tool to inspect the FBG sensor arrays for any damage along the fibre network.

Two RFSG sensors (Tokyo Sokki Kenkyujo Co Ltd. Tokyo, Japan, Type YL-5), each having a gauge length of 5 mm, were used in conjunction with a strain gauge amplifier (National Instruments, SC-2345 signal conditioning instrument). The strain gauge amplifier was equipped with a quarter bridge amplifier module (National Instruments, SCC-SG01) containing two RFSG input channels. The output voltages from the strain gauge amplifier were logged on a PC using a National Instruments data acquisition card (NI PCI-MIO-16E-4).

FE analysis was performed to predict the strains that the fuel pump would experience when it is subjected to static pressure loading (Section FE Modelling). The FE analysis predicted the strain at each of the spatial locations of the FBG sensors on the fuel pump, taking into consideration the orientation of the sensors for measuring the hoop (*X* axis), longitudinal (*Y* axis), and radial strain components (Section FE Modelling). The FE strain data were computed for applied pressures of 50, 70 and 90 bar.

### 3.2. DT Set Up

**Figure 7:**
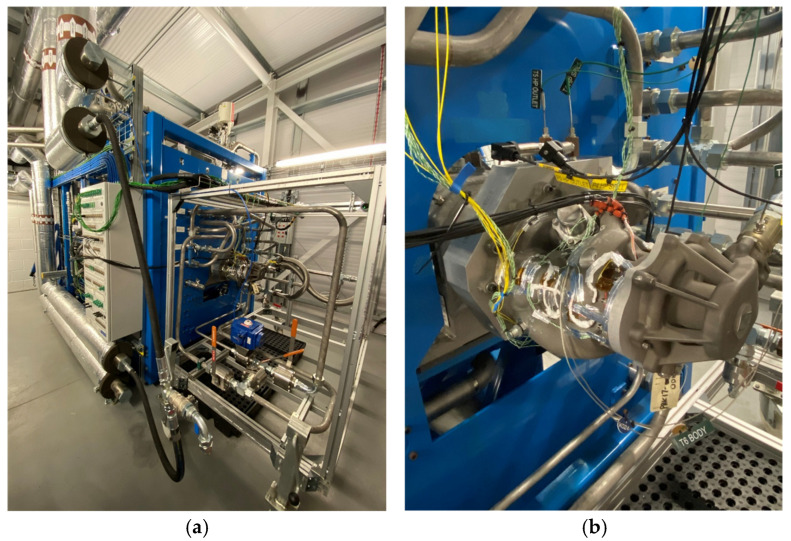
The instrumented fuel pump after installation onto the test rig in Cranfield University’s Thermal Management Systems Facility: (**a**) overview of installation, (**b**) close up of pump. In yellow are optical fibre pigtails from FBG sensors, in green and white are the thermocouple cables, and the multi-coloured cables are from strain gauges.

The instrumented fuel pump was subsequently mounted on the fuel pump test rig located in Cranfield University’s Thermal Management System Facility (TMSF), as shown in Figure 7. The RFSGs were used to provide independent strain measurements for the validation of the FBG strain data, while, as discussed previously, thermocouples were used to compensate for the thermal-induced strain. The TMSF is a designated hazardous area with potentially explosive atmosphere due to the presence of flammable gasses arising from jet fuel, which gives rise to the risk of explosion, and therefore the facility and equipment within it is ATEX rated. The FBG sensors and the RFSGs were connected into their respective data acquisition systems through ATEX isolation barriers.

**Figure 8:**
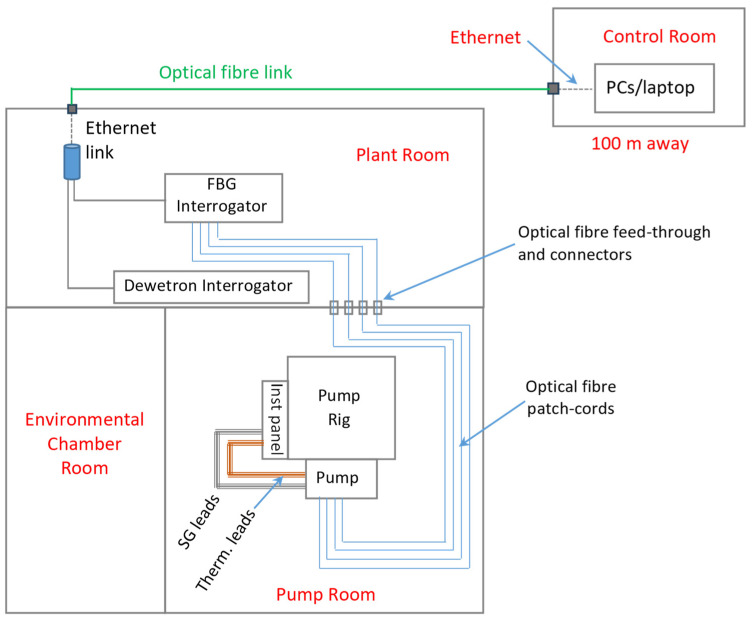
Schematic diagram showing the experimental setup for the fuel pump instrumentation. PC, personal computer; Inst panel, Dewetron Instrumentation Panel; SG, strain gauge; and Therm., thermocouple.

A SmartScan FBG interrogator, situated in the plant room adjacent to the pump room, was used to interrogate the four FBG arrays, which contained a total of 24 FBG sensors, as described in Section 2.4. and in Figure 8. The FBG interrogator was controlled, and the data logged, on a PC located in the control room (about 100 metres away), via an ethernet connection. The data acquisition was performed at a rate of 2.5 kHz, which is its maximum capability.

A Dewetron data acquisition system [45], part of the fuel pump test rig’s infrastructure, was used to interrogate the strain gauges and thermocouple probes using DAQP-STG and DAQP-THERM amplifier modules, respectively, which offer bandwidths of up to 300 kHz. A total of 16 XY T-strain gauge rosettes (type 3/350 XY13; HBM UK), with two orthogonal measuring grids, were used for strain measurement, and were interrogated using Wheatstone quarter and half bridge signal conditioners. The quarter bridge circuit was configured with 4-wire strain gauge connections, which removes the effect of temperature on the resistance of the lead-wire of the strain gauge, and removes the influence of variations in the contact resistance. An added benefit of using the 4-wire system is that long and thin lead-wires can be used without inducing errors in the measurement system. This was particularly useful as the test rig facility pump room is ATEX rated, requiring the data acquisition systems to be located outside of the ATEX zone in the plant room, and therefore a number of sensors required downleads of length up to 10 m and electrical isolation barriers. The half bridge circuit was configured with 5-wire strain gauge connections, allowing compensation for the effects of temperature and variations in contact resistance. The specifications of the strain gauges used in the DT are detailed in Table 1. A total of 13 thermocouple probes were also interrogated by the Dewetron system.

The SmartScan and the Dewetron interrogators were synchronised by referencing their clocks. Each test condition was maintained for a duration of approximately 60 s. Data recording for all sensors was carried out continuously. The DT was carried out under ambient temperature conditions, in which the fuel temperature was not controlled.

## 4. Experimental Procedures

### 4.1. ST Experiment

The pressure applied to the water in the fuel pump was cycled between 0 bar and 90 bar in incremental steps of 10 bar. Data were acquired simultaneously from the 32 FBG sensors and the two RFSG sensors at a rate of 2.5 kHz. Five minutes settling time was allowed at each pressure step before carrying out the data logging over a duration of 5 s. The data were averaged over the two experimental runs, with the error in the strain being given as the calculated standard deviation.

### 4.2. DT Experiment

**Figure 9:**
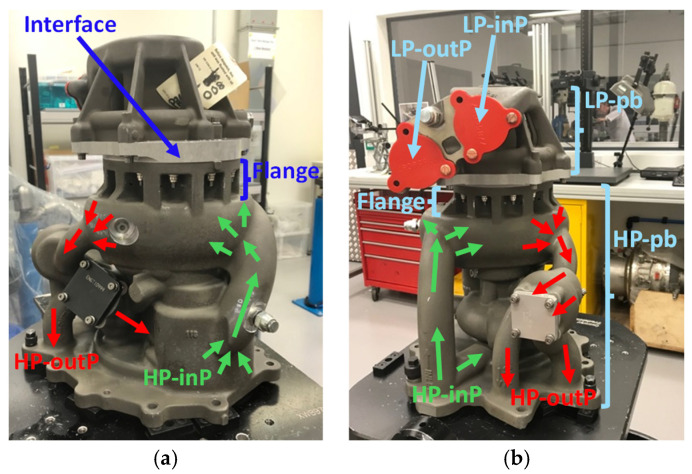
The Rolls-Royce fuel pump: (**a**) side 1, and (**b**) side 2, showing the parts that form the fuel pump body and the pressures in operation during DT. HP-inP: HP inlet pressure, HP-outP: HP outlet pressure, LP-inP: LP inlet pressure, LP-outP: LP outlet pressure, LP-pd: LP pump body, LP-pb: LP pump body. Also shown are the flange and the interface between the LP and HP pump bodies.

Figure 9 shows the two sides of the fuel pump which were instrumented for DT, and also shows the parts that form the fuel pump body and the pressures that were in operation on each of these sides. The fuel pump body consists of a high pressure (HP) pump body and a low pressure (LP) pump body that are joined together at an interface next to the flange of the HP pump body (Figure 9).

The dynamic testing of the pump was carried out using the pump speeds and pressures detailed in Table 2. These are the conditions that the pump is operated at during the *Production Acceptance Test* that all of the pumps are subjected to prior to being fitted to the engine. The higher speed and pressure conditions are representative of the high-power take-off conditions. As the fuel temperature was not controlled, changes in the temperature were due mainly to the work being done on the fuel from the pressuring action on the fuel which, coupled with the recirculating flow, led to larger increases in the temperature of the fuel. The pump speed and outlet pressure were varied during a continuous run of the pump, dwelling at each condition for approximately 60 s. The pressure measurement at the outlet of the high-pressure stage of the fuel pump is denoted as HP. Data were acquired continuously at a rate of 2.5 kHz and averaged over a period of 1 s. The experiment was repeated four times.

### 4.3. The Conditions for Comparing ST and DT

While the results of the ST and DT experiments would not be expected to be identical, they may still be comparable under certain conditions. The ST and FE case allow comparison with DT in circumstances where both the pump temperatures and the applied pressure levels are similar, allowing a more comprehensive analysis and understanding of the DT results than would otherwise be the case.

The experimental conditions that are considered likely to affect the quality of the comparison are the temperature of the pump, the pressure distribution, and the vibration. During DT, the action of pressurising the fuel is doing work on the fuel, causing the fuel temperature to increase which, coupled with the recirculating flow, leads to larger increases in the temperature of the fuel. This change in the fuel temperature, and thus that of the pump, would impact on the expansion of the pump body and thus introduce additional strain on the fibre optic strain sensors. In the work presented here, the influence of temperature on the fibre optic strain measurements was compensated from data obtained experimentally by thermocouples, thus allowing for the comparison of data obtained during ST and DT (Section Principles of FBG Sensors). The influence of vibrations induced in the fuel pump by the rig to which the pump is attached during DT, and by the moving internal components such as the gears—absent in ST—are considered to be minor and this will likely appear as an increase in the noise level in the strain measured during DT. In this work, the influence of transient strains, more likely occurring in DT, were minimised by recording the data at the steady state in both tests.

The pressure distribution across the pump during the two tests will differ. During ST, the LP and HP pump bodies of the fuel pump body have the same pressure values, which is the HP outlet pressure, as the whole fuel pump body was flood-filled with water, whereas during DT there is pressure distribution over the whole fuel pump body which is dictated by the active fuel pumping systems that are operative. During DT, the HP inlet pressure to each gear stage within the HP pump body is lower than the HP outlet pressure. Since in ST the whole fuel pump body is flood-filled with the HP outlet pressure, it follows that for comparable HP outlet pressures for ST and DT, there are likely to be differences between the strain data of ST and DT where the sensors are located in and around the HP inlet pressure regions, while the sensors located around the HP outlet pressure regions (Figure 9) are likely to be closer in value. Within the LP pump body, the pressure increases outward from an LP inlet pressure to the pressure levels that closely approach the levels of the HP inlet pressure at the interface of the LP pump body with the HP pump body (Figure 9). This pressure is therefore likely to influence the strain measurements by the sensors that are located around the flange of the HP pump body to which the LP pump body attaches, which will likely lead to differences between ST and DT strain measurements. Generally, the comparison between the data of ST and DT is likely to be closer in value where the sensors are located around the HP outlet pressure regions of the HP pump body, whereas differences will likely be where the sensors are located around the HP inlet pressure regions and also where the sensors are located at the flange of the HP pump body (Figure 9).

#### FE Modelling

An FE model of the fuel pump, comprising approximately 750,000 elements and 1.2 million nodes, was built and run in ANSYS Workbench v16.2. The purpose of this model was to assess the structural capability of the HP pump body only and so did not include the LP pump body or the pump internal components, which had to be represented by characteristic loads (Figure 9). As yielding was not expected, the model used linear material properties, while non-linear contact between the modelled components was assumed. The FE model was based on a nominal pump geometry and doesn’t take into account variations in the geometry and material thickness that are inherent in the casting process used to fabricate the pump body.

The modelled strains at the locations of the FBGs were determined from the predicted displacements of the nodes corresponding to each end of the FBG. Given the small magnitude of the nodal displacements, and therefore the strains, this process was sensitive to the interpretation of the sensor location within the model, and therefore the selection of the most appropriate nodes to use. Such sensitivity in the choice of nodes for the FBG, together with the possible variation in the pump geometry and material thickness, meant that the predicted strains obtained from the FE model will contain a level of uncertainty. The magnitude of the uncertainty has not been determined.

## 5. Results

Generally, the FBG sensors and the strain gauges compared in this report were located at the same positions on the statically and dynamically pressurised Rolls-Royce fuel pumps, allowing their *direct* comparison. In some cases, the comparison is for situations where the sensors are in neighbouring locations, in which case the comparison is classed as *indirect*. FE outputs at sensor positions determined by the FBG and strain gauge locations for the ST are also used in the comparisons. The FE data are the outputs from a modelling package that was developed in-house (Rolls-Royce), which predicts the deformation of the HP pump bodies and their internal gears and bearings under given operating pressures.

### 5.1. Data Processing for the DT

**Figure 10:**
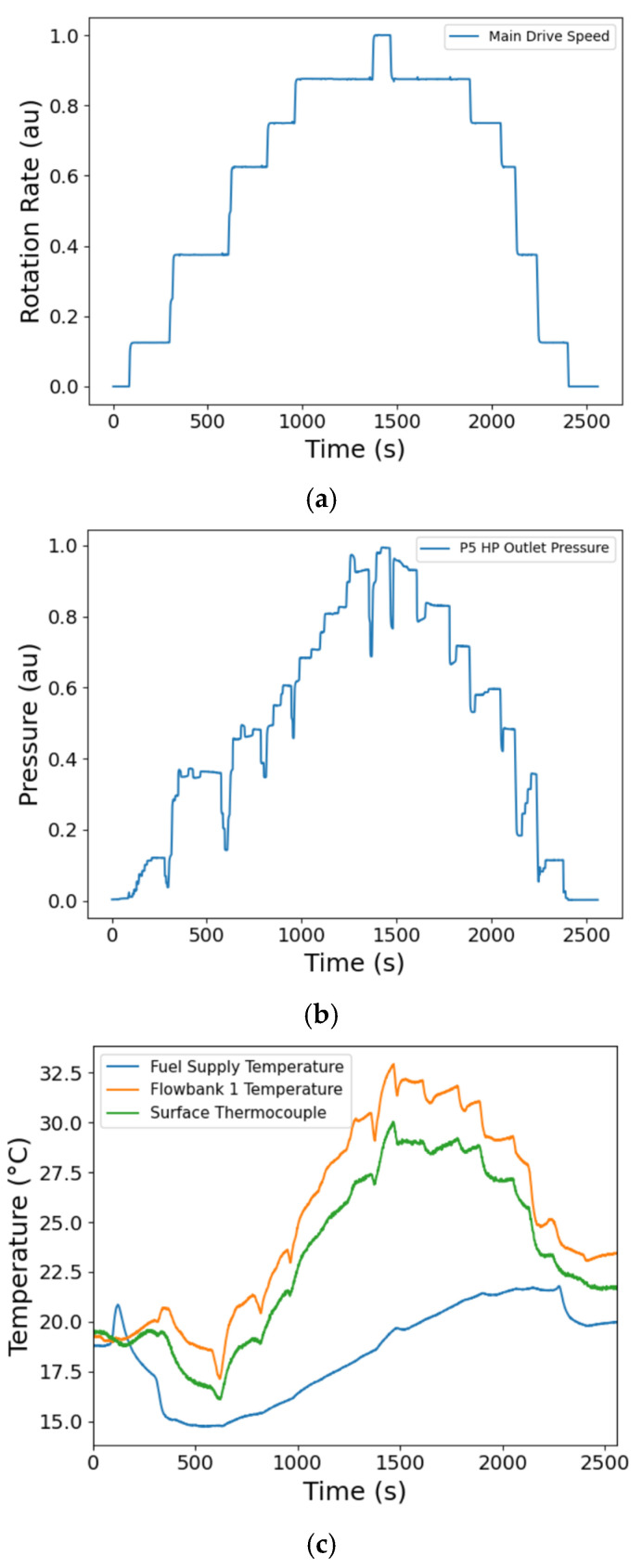
The rig operating conditions: (**a**) the pump speed, which is given as a percentage of the maximum rotation rate, (**b**) the HP outlet pressure, which is given as a percentage of the maximum pressure, and (**c**) the rig temperatures recorded during the DT at ambient conditions for the first run. A thermocouple is attached to the surface of the fuel pump. au—arbitrary units (for normalised data).

Figure 10 shows the significant rig operating conditions during the first experimental run that were recorded by the sensors that are integral to the thermal management systems facility, and logged on a Dewetron data acquisition system. For commercial reasons, the pump speed, shown in Figure 10a, is provided as a percentage of the maximum rotation rate, and the HP outlet pressure, shown in Figure 10b, is given as a percentage of the maximum pressure. The data presented in Figure 10 were recorded as the operator set the rig operation conditions to satisfy the test schedule detailed in Table 2, a process that involved control of the pump speed and small adjustments to pressure regulating values.

A sharp decrease in measured pressure is observed in Figure 10b due to the opening of the pressure regulating valve prior to an increase of speed, to prevent an overpressure arising upon setting the next test condition in Table 2. The maximum change in the temperature (Figure 10c) is seen in the Flowbank, which is a metered flow line from the pump rig to the fuel pump, where the temperature is measured by a rig-installed thermocouple. On the other hand, the fuel supply sensor, which monitors the temperature of the fuel reservoir, shows the smallest temperature change, which would be expected as this is heated only by the recirculation of the fuel. The surface temperature was monitored by a thermocouple located near the outlet of the HP stage of the fuel pump, showing close comparison to the Flowbank temperature (Figure 10c). Steps can be observed in the temperatures of the thermocouple and the Flowbank that correspond to the steps in the rotation rate of the fuel pump, showing that the action of pressurising the fuel is directly related to the heat generated in the fuel as it circulates.

**Figure 11:**
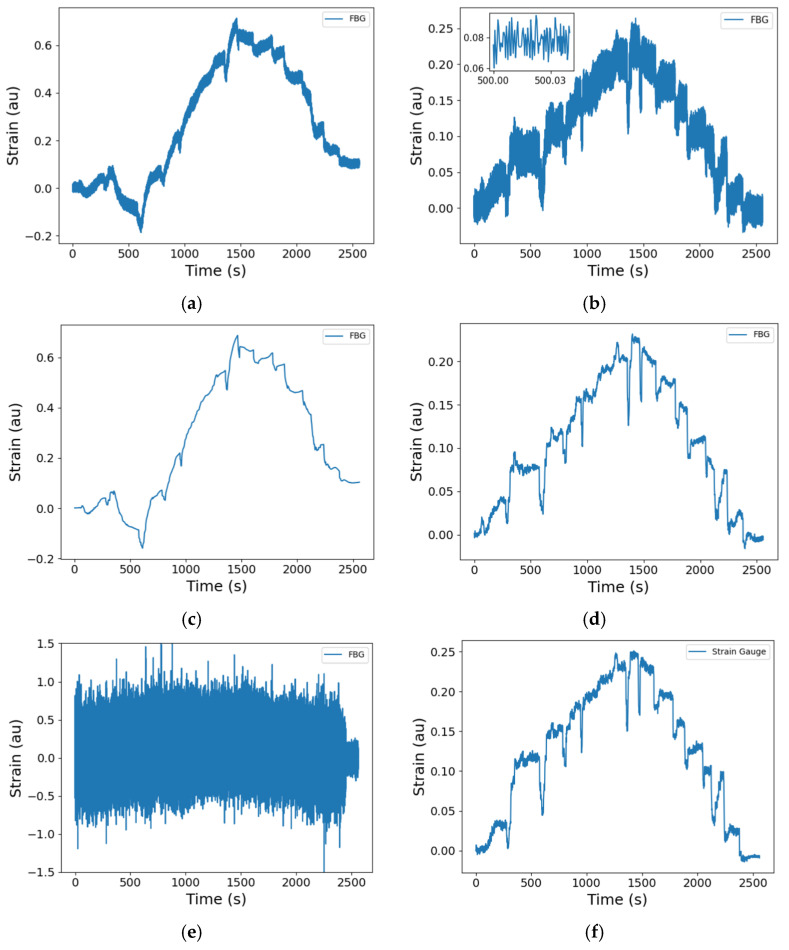
Raw strain data illustrating the typical noise levels associated with the measured signals: (**a**) FBG at full data rate (2.5 kHz), (**b**) temperature compensated FBG at full data rate, (**c**) FBG at 1 Hz data rate, (**d**) temperature compensated FBG at 1 Hz data rate, (**e**) strain gauge at full data rate (2.5 kHz), and (**f**) strain gauge at 1 Hz data rate. The presented strains are all normalised to the maximum strain that was measured during the testing. au—arbitrary units (for normalised data). Raw strain data are the apparent strain determined from the measured FBG wavelength shift before compensation for the influence of temperature.

The noise floor for the FBG measurement is determined by the SmartScan FBG interrogator, and typical standard deviations are 30 nε/√Hz [26]. The calculated strains presented in this work are averaged, producing an equivalent downsampling rate of 1 Hz from an acquisition rate of 2.5 kHz. For commercial reasons, the presented strains in this paper are all normalised to the maximum strain that was measured during the entire testing campaign, taken from data downsampled to 1 Hz to avoid the influence of noise spikes such as those evident in the unfiltered strain gauge data shown in Figure 11e. The strain data shown in Figure 11 are representative of the recorded time series, and do not show the data from the sensors or test where the maximum strain was observed. The comparison of the raw strains, at the full 2.5 kHz data rate (Equation (4)), measured by an FBG with the neighbouring RFSG, are shown in Figure 11. Raw strain, in this paper, describes the apparent strain derived from the total FBG wavelength shift, that is, before the compensation for temperature (or thermal strain) was undertaken. The RFSG measurements are affected by electrical pickup from within the test section produced by electromagnetic interference generated by operating the test rig, as shown by the diminished signal-to-noise ratio in Figure 11e when compared to that of the FBG measurement (Figure 11a) for the signals at the full data rate of 2.5 kHz. The temperature compensated FBG strain at the full data rate (Figure 11b) depicts increased noise levels when compared to the raw FBG strain at full data rate (Figure 11a), mainly because the thermocouple data used to compensate for temperature were acquired at a lower data rate of 5 Hz, which was a limitation of the TMSF data acquisition system. The temperature data were therefore interpolated to match the full data rate before being used to compensate the raw FBG strain at the full data rate. The inset in Figure 11b reveals the underlying sinusoidal oscillations in the data, which are related to the rotation of internal components within the pump (Section 5.3).

To allow the relationship between the fuel pressure and the measured strains to be investigated, a script was written in Python 3.11 to identify the durations over which the HP pressure was in the steady state (Figure 10b). The average signal of each RSFG and FBG at each steady state condition was calculated and plotted as a function of the HP outlet pressure. Typical time series are shown in Figure 10 and Figure 11, where the steps and steady state conditions are apparent. To explore the dynamic characteristics of the fuel pump, spectrograms of the raw wavelength shifts, Δ*λ_B_*, of the FBGs, described in Equation (2), were determined by calculating the short-time Fourier transform of the data time series to localise the frequency components excited in time.

### 5.2. Comparison of DT and ST

**Figure 12:**
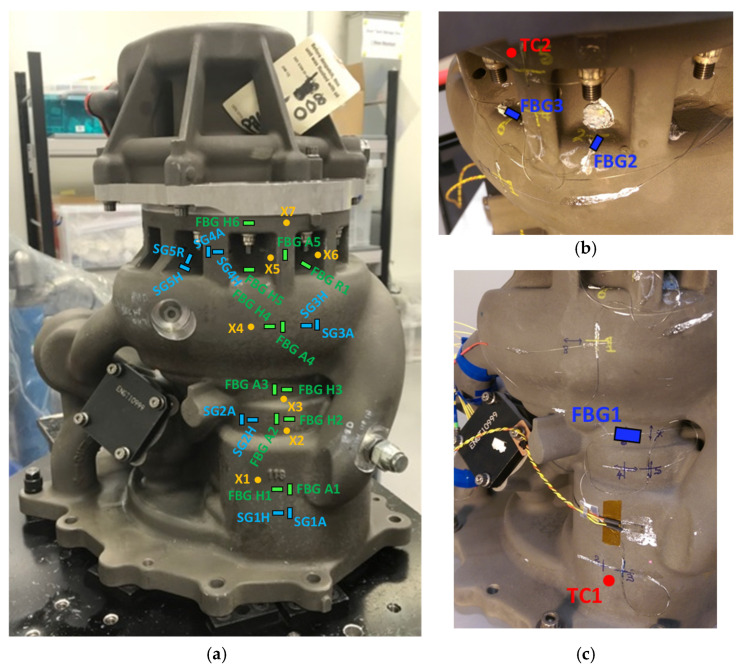
Fuel pump side 1 showing the positions and orientations of the FBG sensors and RFSGs for (**a**) DT, and (**b**,**c**) the FBG sensors that are being compared with the ST using a hydraulic hand pump performed on a separate fuel pump. FBG A1–FBG A5, axial FBGs; FBG H1–FBG H6, hoop FBGs; FBG R1, Radial FBG; X1–X7, thermocouples; SG1A–SG4A, axial strain gauges; SG1H–SG5H, hoop strain gauges; SG5R, radial strain gauge; TC1, bottom thermocouple; TC2, top thermocouple.

The sensors that are the subject of the comparison between the ST and DT are detailed in Table 3 and their locations and orientations are shown in Figure 12. The FBG data are compensated for the influence of temperature. Figure 12a shows the marked positions and orientations of the sensors before installation for the fuel pump instrumented for DT. In the labelling of the sensors, H, R and A refer to the hoop, the radial and the axial (longitudinal) directions on the fuel pump, respectively. The FBG sensors oriented with the axial, hoop, and radial directions of the fuel pump are marked as FBG A1–FBG A5, FBG H1–FBG H6, and FBG R1, respectively. The strain gauges oriented with the axial, hoop, and radial directions of the fuel pump are marked as SG1A–SG4A, SG1H–SG5H, and SG5R, respectively. The positions of the thermocouple probes are indicated as X1–X7 (Figure 12a). These probes were required to compensate for thermal strains in the FBG measurements, as discussed in Section Principles of FBG Sensors. Figure 12b,c indicate the positions and orientations of those sensors on the ST fuel pump which are used in the comparisons with the DT. The locations of two thermocouple probes used in the ST are indicated as TC1 and TC2 (Figure 12b,c).

**Figure 13:**
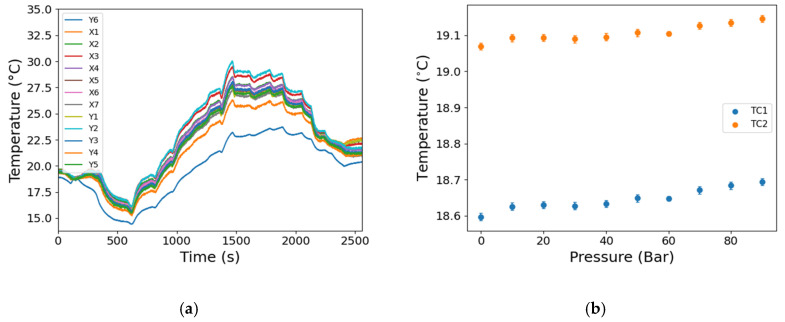
Temperature measurements from the thermocouples attached to the surface of the fuel pump during (**a**) the DT and (**b**) the ST. The locations of the thermocouples are described in Figure 12 for side 1 and the thermocouples Y1–Y5 for side 2. TC1, thermocouple at bottom of pump; TC2, thermocouple at top of pump for ST.

Figure 13 shows the surface temperature of the fuel pump measured by thermocouple probes distributed over the pump surface for the DT (Figure 13a) and ST (Figure 13b). The fuel pump temperature changes by approximately 15 °C during the DT (Figure 13a), mainly due to the work being done on the fuel from the pressuring of the fuel and the recirculation of the fuel through the fuel reservoir, the temperature of which is not controlled. The measured temperature varies across the pump, revealing the differences in the heat conduction at the location of the thermocouples, which is a function of the local interior and exterior structure of the fuel pump. There is a noticeable decrease in temperature during approximately the first 600 s, which can be attributed to the initial flow of the relatively cold fuel from the reservoir (Figure 13a). In contrast, the temperature only changed by approximately 0.1 °C over the duration of the ST experiment (Figure 13b).

**Figure 14:**
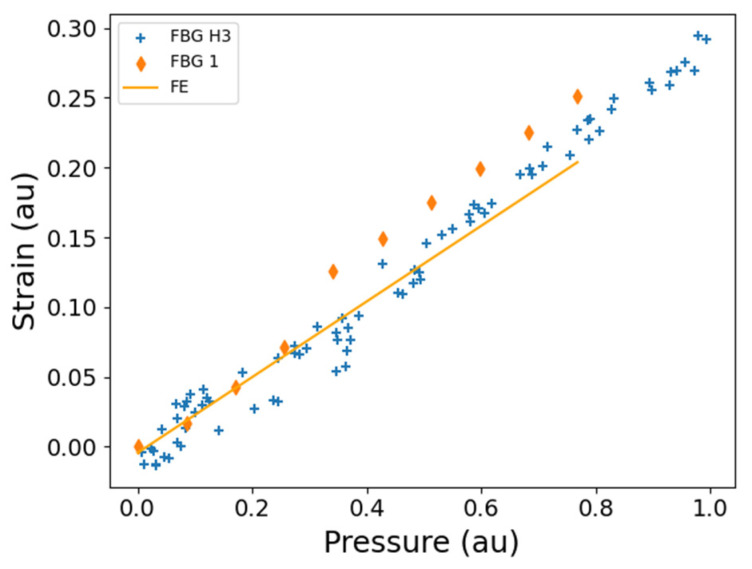
The FBG measurements plotted as a function of HP outlet pressure during DT for side 1 of the fuel pump (Figure 12). Plotted on the same graph is the FE analysis and FBG measurements from ST on a similar fuel pump that was pressurised with water. FBG H3 (DT), hoop FBG (DT). FE (ST), FE analysis performed for the position of FBG1; FBG1 (ST), hoop FBG (Figure 12). The presented strains are all normalised to the maximum strain that was measured during the testing. au—arbitrary units (for normalised data).

The strain measured by FBG H3 during the DT is in close agreement with both the strain measured in the ST by FBG1, and with the predictions of the FE model (Figure 14). The FBGs strain data reveal two strain regimes (separated by a step change in strain) that are depicted by the pressure ranges of ~0–0.4 au and ~0.4–1.0 au, and within these regimes the strain varies linearly with respect to pressure (Figure 14). This effect, more pronounced in ST than in DT, may be attributed to the characteristic behaviour of the pump at the specific spatial location of the FBG sensors (Figure 12). The FBG strain data in DT exhibit some hysteresis in the strain regime of ~0–0.4 au, as observed in the spread of the data points of the FBG (Figure 14). The strains measured by the two FBGs and the strain predicted by FE are in agreement within the pressure regime of ~0–0.4 au, while the average of the difference in the strain between the FBG data of ST and DT is approximately 0.02 au within the pressure regime of ~0.4–0.8 au. The two FBG sensors that are being compared are just below the bulge around the middle of the fuel pump (Figure 12a,c). This location of the sensors on the pump is where agreement is expected as the pump conditions are similar for both ST and DT experiments as discussed in Section 4.3. These sensors are located around the HP outlet pressure region where good agreement is likely expected as discussed in Section 4.3 (Figure 9).

**Figure 15:**
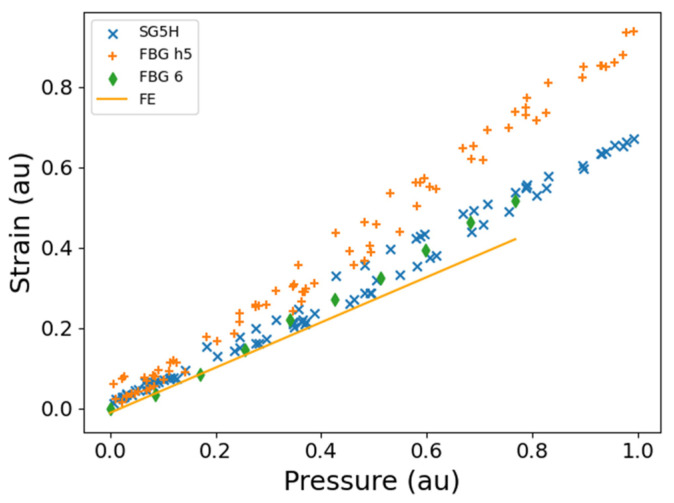
The FBG and strain gauge measurements as a function of HP outlet pressure for DT for side 1 of the fuel pump (Figure 12). Plotted on the same graph is the Finite Element analysis and FBG measurements from the ST of a similar fuel pump that was pressurised with water. DT: SG5H, strain gauge 5 hoop; FBG H5, hoop FBG for DT (Figure 12a). FE (ST), FE analysis performed for the position of FBG6; FBG6 (ST), hoop FBG (Figure 12b). The presented strains are all normalised to the maximum strain that was measured during the testing. au—arbitrary units (for normalised data).

In Figure 15, the data from FBG H5 and strain gauge SG5H recorded during the DT are compared. The strains diverge for pressures greater than approximately 0.5 au, possibly due to the spatial displacement between the sensors (Figure 12). Strains measured by FBG6 and predicted by FE analysis for the ST are in good agreement with the measurement from FBG H5. These sensors are located at the flange on the HP pump body (Figure 9), nearest to the interface between the HP and LP pump bodies (Figure 12). The difference in the results for ST and DT is likely here because the sensors are located at the HP inlet pressure region which is also at the interface between the two pump bodies, as discussed in Section 4.3. In ST, because the fuel pump body is flood-filled with water, the pump body has the same pressure as the HP outlet pressure which will be comparable only to HP outlet pressure in DT, which means that the sensors in such locations are more likely to show similar values (Section 4.3).

**Figure 16:**
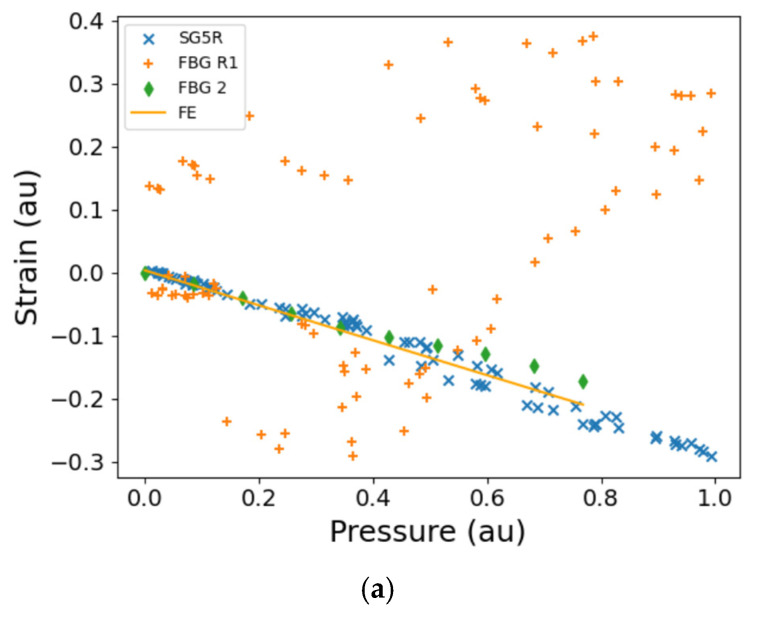

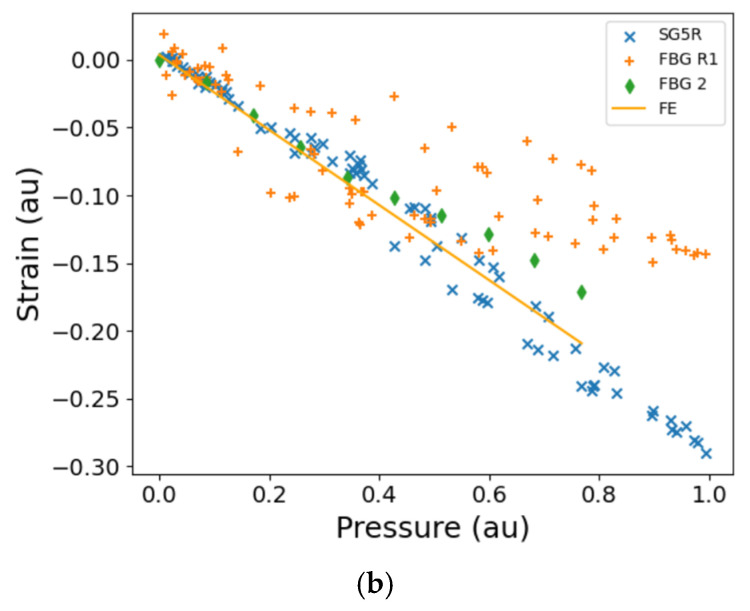
The FBG and strain gauge measurements as a function of HP outlet pressure for DT (**a**) before FBG temperature compensation, and (**b**) after FBG temperature compensation for side 1 of the fuel pump. Plotted on the same graph is the Finite Element analysis and FBG measurements from the ST of a similar fuel pump that was pressurised with water. SG5R (DT), strain gauge 5 Radial; FBG R1 (DT), Radial FBG (Figure 12a). FE (ST), FE analysis performed for the position of FBG2; FBG2 (ST), Radial FBG (Figure 12b). The presented strains are all normalised to the maximum strain that was measured during the testing. au—arbitrary units (for normalised data).

The sensors being compared here (Figure 16b) are located at the flange on the HP pump body (Figure 9), nearest to the interface between the HP and LP pump bodies (Figure 12). Discrepancy is likely between the results of ST and DT here, as discussed in the paragraph above and in Section 4.3. Data from strain gauge SG5R during the DT show close agreement with the FE calculations for the ST (Figure 16b). The measured strain from FBG R1 is in relatively close agreement with data from SG5R for the DT after temperature compensation in the FBG data (Figure 16b), whereas the two sensors are not correlated before FBG temperature compensation (Figure 16a). This shows that the temperature compensation methodology employed in this paper was effective. The difference between FBG R1 and SG5R is expected, given that the inset sections of the pump’s ribbed end (Figure 1a) in which the sensors are located are separated by another inset section (Figure 12), combined with the influence of the difference in the temperature at the two locations (Figure 13). From the ST, the data from FBG2 are in good agreement with the strain measured by FBG R1 in the DT (Figure 16b).

**Figure 17:**
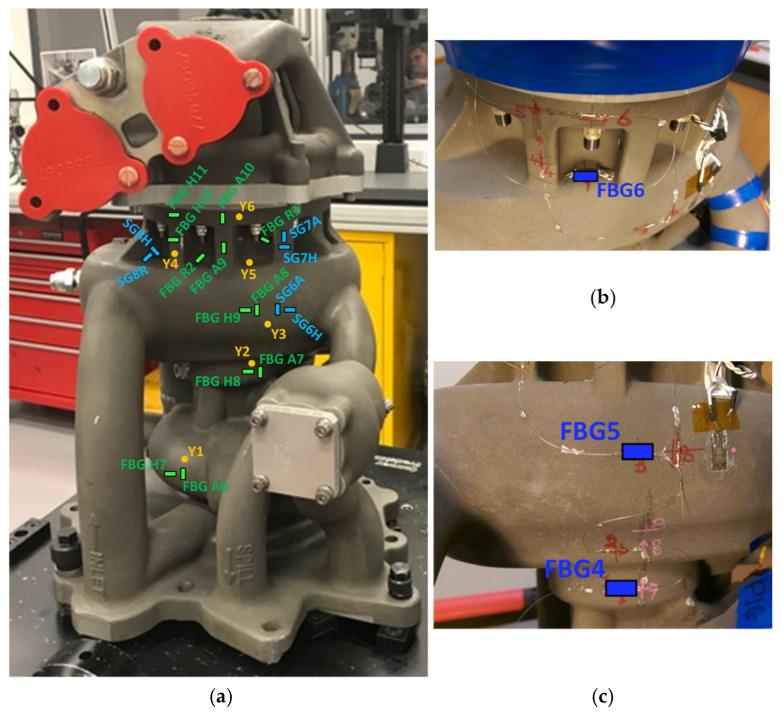
The positions and orientations of the FBG sensors and strain gauges on side 2 of the fuel pump for (**a**) DT, and (**b**,**c**) the FBG sensors that are being compared with the ST using a hydraulic hand pump performed on a separate fuel pump. FBG A6–FBG A10, axial FBGs; FBG H7–FBG H11, hoop FBGs; FBG R2 and FBG R3, Radial FBGs; Y1–Y6, thermocouples; SG6A and SG7A, axial strain gauges; SG6H–SG8H, hoop strain gauges; SG8R, radial strain gauge.

Table 4 and Figure 17 detail the sensors from the DT and ST, installed on side 2 of the fuel pump. The fuel pump is non-symmetric, is complex in shape, and the presence of gears and their specific positioning inside means that the pump body itself has a complex configuration. These characteristics of the fuel pump necessitated the need to instrument as much surface area as possible, with side 1 and side 2 together providing the needed sensor coverage.

**Figure 18:**
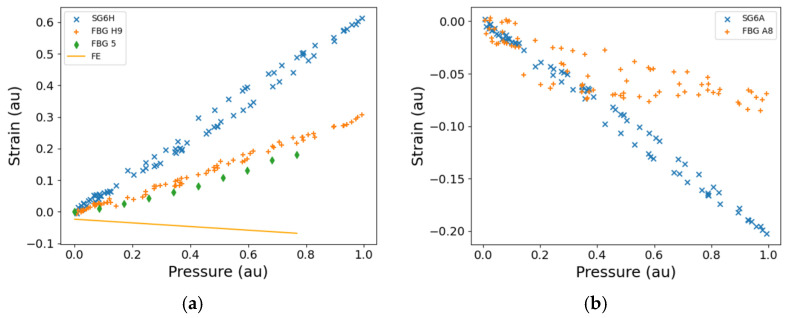
The FBG and strain gauge measurements as a function of HP outlet pressure for DT for (**a**) hoop strains, and (**b**) axial strains for side 2 of the fuel pump (Figure 17). Also plotted in (**a**) is the Finite Element analysis and FBG measurements from the ST of a similar fuel pump that was pressurised with water. SG6H (DT), strain gauge 6 hoop; SG6A (DT), strain gauge 6 axial; FBG H9 (DT), hoop FBG (Figure 17a); FBG A8 (DT), axial FBG (Figure 17a). FE (ST), Finite Element analysis performed for the position of FBG5; FBG5 (ST), hoop FBG (Figure 17c). The presented strains are all normalised to the maximum strain that was measured during the testing. au—arbitrary units (for normalised data).

The vicinity of the sensor location here is such that the flange is directly above it, while to the left side and to the right side respectively are the HP inlet pressure and the HP outlet pressure regions for the case of DT (Figure 9). During DT, the sensors are therefore likely to experience simultaneously the influences of the HP inlet pressure, the HP outlet pressure, and the strains in the flange that attaches the HP pump body to the LP pump body (Figure 9). The data from the sensors in this location will thus likely be sensitive to the sensor orientation. Given the proximity of these pressure and strain regions to the sensor location discussed here, the nature of the agreement between the results of ST and DT is likely dependent on the precise sensor location (Section 4.3). Strain measurements from strain gauge SG6H obtained during DT are larger than the strains from FBG H9 (Figure 18a). The strains measured by FBG H9 in DT are in good agreement with the strain measured in ST by FBG5 (Figure 18a). The difference in the strains measured by the FBGs and the strain gauge could arise from the spatial separation of the sensor locations, approximately 40 mm (Figure 17) and the likely resulting variation in the temperature (Figure 13). The FE predicts a compressive load and is not in agreement with the experimental data. Since, at this location, the hoop strains recorded by both FBG H9 and SG6H are positive (Figure 18a) and the axial strains recorded by both FBG A8 and SG6A are negative (Figure 18b), it suggests that a small misalignment of the orientation of the nodes of the FE model could result in an appreciable discrepancy between the hoop strain calculated by the FE and the strains measured by the FBG and the RFSG. In order to determine small strains of less than 100 me from the FE model, small values of nodal displacement have to be extracted (in the relevant direction) from the locations that are approximately representative of either end of the FBG, as discussed in Section FE Modelling. This procedure is known to be sensitive to the interpretation of the location of the sensor.

### 5.3. Frequency Response of the Fuel Pump

**Figure 19:**
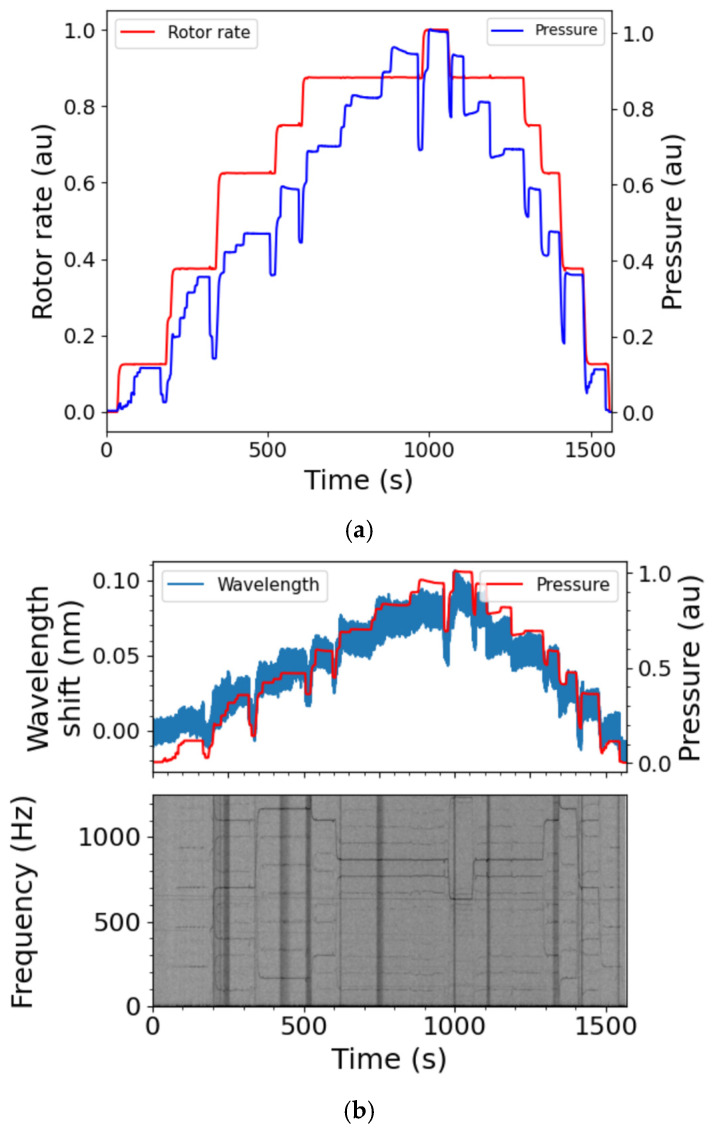
The spectrogram of FBG H3 (Figure 12a) for the DT of the fuel pump: (**a**) the pump speed and fuel pressure as a function of time, and (**b**) spectrogram of FBG H3 as a function of time. The inset above plots the wavelength shift of FBG H3 and fuel pressure obtained at the full data rate of 2.5 kHz. The pump speed is given as the percentage of the maximum rotation speed while the pressure is given as the percentage of the maximum HP outlet pressure. au—arbitrary units (for normalised data).

The frequency response of the fuel pump was investigated by creating a spectrogram from the FBG time series recorded during DT. This allowed the frequency characteristics of dynamic events to be localised in time. Figure 19 shows the spectrogram of the FBG data from FBG H3 (Figure 12a). There are clear features that follow the pump rotation frequency. During the transitions when the pump rotation speed was being either ramped up or ramped down, there is evidence of broad-spectrum excitation. The observed features can be further understood with consideration of the pump’s design and features in relation to the installed FBG sensors. With reference to Section 2.1. and Figure 1, the FBGs are located as follows: FBG H1 is at the location of the small displacement gear, FBG H2 is located in the space between the two gear stages, FBG H3 is bonded over the location of the large displacement gear stage, FBG H4 is located in the space between the large displacement gear and the centrifugal impeller, and FBG R1 is at the location of the centrifugal impeller.

Table 5 is a summary of the evolution of the dominant frequency in the spectrogram of the FBG data from FBG H3 (Figure 19), in response to the rotation rate of the pump as it was changed from 0 to maximum and back to 0 rpm over the total duration of 1 600 s. The table also shows the expected pump frequency by considering the presence of a 14-tooth gear. The frequency measured by FBG H3 is 14 times the pump rotation rate, in agreement with the number of teeth in the gear. As shown in the table, the maximum expected frequency is 1866.7 Hz. The SmartScan instrument that was used to interrogate the FBG sensors has a maximum acquisition rate of 2 500 Hz, which allows a maximum measurable frequency of 1 250 Hz, according to the Nyquist theorem. The entries in bold text in Table 5 represent the frequency components which do not obey the Nyquist theorem, which are aliased back into the Nyquist frequency range. However, it is trivial to determine the true frequencies by subtracting the aliased frequency from the Nyquist limits and then adding the result to the Nyquist limit. In addition to the dominant spectrogram frequencies recorded in Table 5, the harmonics of these frequencies (Figure 19b) were also seen in the five FBGs described in this section, FBG H1–FBG H4 and FBG R1. The harmonic frequencies are dominant for FBG H3, FBG H4 and FBG R1. As discussed above, on one end the large displacement gear pump directly drives the small gear pump, while on the other end it directly drives the centrifugal impeller [31]. These three FBG sensors installed on side 1 are located on the surface and in the segment of the fuel pump where the large displacement gear is driving the centrifugal impeller, and it seems that the impeller is having an additional impact on these sensors (Figure 19b).

## 6. Discussion

The dynamic pressure testing has successfully demonstrated the use of FBG sensors to measure the distribution of surface strain on an aircraft fuel pump, providing both time-averaged and dynamic measurements, up to a frequency of 2.5 kHz, with high signal integrity comparable to that obtained from the static pressure tests performed in a laboratory. Typical noise standard deviations of 1.5 µε over the 2.5 kHz bandwidth were obtained, corresponding approximately to 30 nε/√Hz, for FBG strain data measured with the SmartScan FBG interrogator [26]. These experiments have shown that ancillary components to the fuel pump induce additional frequency components apart from the rotation rate of the fuel pump. The experiments have shown the importance of temperature compensation. The knowledge gained from the static pressure tests proved vital in the enabling of optimised fibre routing, in providing effective fibre protection, and in facilitating the identification of critical positions on the fuel pump for locating the sensors.

The poor signal to noise ratio of the electrical strain gauge signals, which might be caused by pickup from the long downleads, was such that the data had to be averaged to an equivalent data rate of 10 Hz to allow visualisation of the strain. The data from the fibre optic sensors at the full acquisition rate (2.5 kHz) allowed the slowly varying strains resulting from control inputs to be observed, and, via appropriate spectral analysis, observation of the vibration characteristics of the pump, which could not be observed from the strain gauge data due to the noise. The vibrations were dominated by a frequency produced by the combined effect of the pump rotation rate and the number of teeth in the pump gear, and the harmonics of this frequency. Good agreement was found at many locations of the sensors on the pump between the data from the strain gauge and the neighbouring FBG for the DT. The FE analysis and the FBG and strain gauge measurements obtained from the prior ST, also performed at ambient conditions, were also in good agreement with the measurements from the sensors in DT at the sensor locations where pressure levels in the fuel pump were similar for both tests.

The use of thermocouple probes to perform the compensation for the thermal strain in the FBG measurement poses some challenges, especially as the probe cannot be exactly co-located with the FBG, which can compromise the fidelity of the temperature compensation, particularly if there are significant temperature gradients. The spatial separation of the sensors can also introduce the possibility of thermal lag between the FBG and thermocouple measurements when there are dynamic temperature changes. Thermal lag could also arise from different thermal time constants of the sensors, while issues with synchronising the different acquisition systems could introduce errors when compensating for the temperature. If high temperatures are involved, and/or if the experiment is performed over a wide temperature range [41], then the FBG should ideally be calibrated in situ in order to effectively compensate for the temperature, as the thermal loading of the FBG depends on the heat conduction and the thermal expansion coefficient of the substrate, especially for a structure so complex in shape.

The simple approach to temperature compensation applied to the FBGs in this paper, where the temperature fluctuations were typically less than 10 °C for the duration of the DT, functioned well. Previous studies have shown that the temperature coefficients of an FBG are quadratic, where a temperature swing of, for example, 150 °C, can lead to a non-linear temperature deviation of approximately 4 °C [41]. If the tests were undertaken at elevated dynamic temperatures, then a different temperature compensation method would be needed, as the complexity of the internal and external structure of the fuel pump will lead to the FBG and its associated thermocouple probe experiencing different temperatures due to the thermal lag. The FBG temperature compensation is also affected by the thermal expansion of the fuel pump, but as the measurements were performed at ambient temperature, a nominal thermal expansion coefficient for aluminium was used in the calculations. There are no reported measurements of temperature-compensated strain by FBG sensors on turbomachinery components, but some researchers have performed feasibility studies with fibre optic sensors to measure temperature only [29,46]. As an outcome from these results, further work will explore the deployment of a new fibre optic sensor design based on a tilted FBG [47] to also measure temperature simultaneously with the measurement of strain distribution and vibration in order to improve the accuracy of temperature discrimination in the strain measurements.

This work has presented a new experimental approach to the measurement of a complex-shaped and complex-functioning turbomachinery structure in a harsh and explosive environment, whereby the structure consists of subsystems that include the fuel pump structure, the rotating systems consisting of gears and impellers, and the fuel flow under high applied pressure. Therefore, the instrumented structure is impacted by the interaction of the fluid dynamics of the fuel, the rotational dynamics, and the thermodynamics of the fuel, gears, impellers and the complex-shaped pump body. The reported measurement applications in the literature generally do not encounter integrated subsystems; such examples include pipe integrity monitoring [22], blade vibration detection [18], advanced composites monitoring [32], monitoring of rotor blade loading [24,25], structural health monitoring of civil structures [27], and applications to oil and gas production [22,23]. In addition, this work has for the first time demonstrated the ability to access externally the measurements of strain distribution and vibration for applications to condition monitoring without the need for disassembling of the subsystems, unlike previous reports in other applications [30].

## 7. Conclusions

With the ever-increasing demands to improve the fuel burn efficiency of aero gas turbines, fuel system pressures and temperatures are continually rising. This poses challenges for the structural integrity of the fuel pump housing. It also impacts the internal deflections of the fuel pump which, in turn, can adversely affect the volumetric efficiency of the pump. Having a non-invasive way of measuring the strain in the pump housing while the pump is operating allows these transient effects to be quantified and be implemented during the design process which leads to more robust designs of fuel pump.

This paper has demonstrated the successful integration of fibre optic FBG sensors onto a complex gas turbine engine fuel pump surface, a high signal integrity for both laboratory conditions and for the harsh environment of the test rig experiments, and the real-time measurement of the distribution of strain and of vibration. The deployment of the fibre optic sensors and their protective packaging onto the fuel pump survived the rough handling and experimentation in a harsh environment, demonstrating great potential for this aerospace application.

The strain and vibration measurements presented here suggest that fibre optic sensors could play a role in the structural health monitoring of future aircraft in real-time, whereby the strain could provide warning on potential failure to bolts and nuts used to mount the fuel pump structure while the vibration could similarly provide warning related to gear and impeller failure. Our studies are timely, as demonstrated by current research interests into fibre optic interferometric acoustic sensing in gas turbines [48] and into combustion pressure dynamics of turbomachinery [49]. Our work has shown that carefully located externally mounted sensors are sufficient to characterise the dynamics of internal components, which is significant as it is often impractical to strip the fuel pump or turbomachinery components to perform direct measurements [48]. The absence of electrical cabling offers weight saving in comparison with traditional electromechanical sensors.

## Figures and Tables

**Table 1:** The specifications for the XY T-strain gauge rosettes (type 3/350 XY13, HBM UK).

Parameter	Specification
Gauge length	3 mm
Resistance	350 ohms ± 0.35%
Gauge factor	2.07 ± 1.0%
Temperature coefficient of gauge factor	(101 ± 10) × 10^−6^/K; for −10 to +45 °C
Transverse sensitivity	0.3%
Temperature compensation coefficient (Aluminium)	23.0 × 10^−6^/K
Maximum operating temperature	200 °C

**Table 2:** Ambient test conditions for investigating strain distribution of the fuel pump during DT. The loading case is from test condition 1 to 8, while the unloading case runs from 9 to 15. HP, High Pressure, is the pressure measurement at the outlet of the high-pressure stage of the fuel pump. The pump speed is given as the percentage of the maximum rotation speed while the pressure is given as the percentage of the maximum HP outlet pressure.

Test Condition	Pump Speed (%)	HP Outlet Pressure (%)
1	12.5	11.8
2	37.5	35.3
3	62.5	47.1
4	75	58.8
5	87.5	70.6
6	87.5	82.4
7	87.5	94.1
8	100	100
9	87.5	94.1
10	87.5	82.4
11	87.5	70.6
12	75	58.8
13	62.5	47.1
14	37.5	35.3
15	12.5	11.8

**Table 3:** Details of the sensors deployed in the DT and in the ST of the fuel pump, which are the subject of the comparison in this section (see Figure 12). Direct—the compared sensors are in the same spatial locations; Indirect—the compared sensors are in displaced spatial locations.

DT Sensors	Sensor Orientation	Comparison	ST Sensors
FBG H3	Hoop	Direct	FBG1
SG2H	Hoop	Indirect	-
FBG H5	Hoop (groove bottom surface)	Direct	FBG3
SG5H	Hoop (groove bottom surface)	Indirect (adjacent grooves)	
FBG R1	Radial (groove bottom surface)	Direct	FBG2
SG5R	Radial (groove bottom surface)	Indirect (separated by a groove)	

**Table 4:** Comparison of the DT with the ST of the fuel pump for the sensor locations and orientations on side 2 of the pump (Figure 17). Direct—the compared sensors are in same spatial locations, Indirect—the compared sensors are in displaced spatial locations.

DT Sensors	Sensor Orientation	Comparison	ST Sensors
FBG H8	Hoop	Direct	FBG4
SG8H	Hoop (groove bottom surface)	Indirect (adjacent grooves)	FBG6
FBG H5	Hoop (groove bottom surface)	Indirect (opposite pump sides)	
FBG H9	Hoop	Direct	FBG5
SG6H	Hoop	Indirect (adjacent)	

**Table 5:** The change in the dominant spectrogram frequency of FBG H3 as the rotation rate of the pump is changed from 0–maximum–0 rpm, over a total duration of 1 600 s (Figure 19). FBG H3 is bonded to the surface of the fuel pump at the location of the large displacement gear pump for the pump used in these experiments. The entries in bold text represent the frequency components which do not obey the Nyquist theorem, which are aliased back into the Nyquist frequency range.

Control Parameter	FBG Measurement		Fundamental Vibration Frequency
Rotation Rate (Hz)	Approximate Dominant Spectrogram Frequency (Hz)	Multiple of Rotation Rate	Frequency from Pump of 14-Tooth Gear (Hz)
16.7	235	14	233.3
50	700	14	700
83.3	1 170	14	1166.7
100	**1 100**	**11**	1400
116.7	**875**	**7.5**	1633.3
133.3	**630**	**4.7**	1866.7
116.7	**875**	**7.5**	1633.3
100	**1 100**	**11**	1400
83.3	1 170	14	1166.7
50	700	14	700
16.7	235	14	233.3

## Data Availability

For research data or other materials referred to in this paper, please access the Cranfield Online Research Data repository at https://doi.org/10.57996/cran.ceres-2670.

## References

[1] Mevissen F., Meo M. (2019). A Review of NDT/Structural Health Monitoring Techniques for Hot Gas Components in Gas Turbines. Sensors.

[2] Davinson I. (1984). The Applications of Fibre Optics in Gas Turbine Engine Instrumentation. SPIE Fibre Optics ’84.

[3] Davinson I. (1991). Use of optical sensors and signal processing in gas turbine engines. Proc. SPIE.

[4] Atkins R.A., Gardner J.H., Gibler W.N., Lee C.E., Oakland M.D., Spears M.O., Swenson V.P., Taylor H.F., McCoy J.J., Beshouri G. (1994). Fiber-optic pressure sensors for internal combustion engines. Appl. Opt..

[5] Xia H., Byrd D., Dekate S., Lee B. (2013). High-Density Fiber Optical Sensor and Instrumentation for Gas Turbine Operation Condition Monitoring. J. Sens..

[6] McCord R.M. Engine Condition monitoring using Fibre optic Probes. Proceedings of the ASME 1980 International Gas Turbine Conference and Products Show.

[7] Fischer A. (2017). Imaging flow velocimetry with laser Mie scattering. Appl. Sci..

[8] James S.W., Tatam R.P., Elder R.L. (1997). Design considerations for a three dimensional fiber optic laser Doppler velocimeter for turbomachinery applications. Rev. Sci. Instrum..

[9] García I., Beloki J., Zubia J., Aldabaldetreku G., Illarramendi M.A., Jiménez F. (2013). An Optical Fiber Bundle Sensor for Tip Clearance and Tip Timing Measurements in a Turbine Rig. Sensors.

[10] Vakhtin A.B., Che S.J., Maasick S.M. Optical Probe for Monitoring Blade Tip Clearance. Proceedings of the 47th AIAA Aerospace Sciences Meeting including the New Horizons Forum and Aerospace Exposition.

[11] Jenkins T.P., Allison S.W., Eldridge J.I. (2013). Measuring gas turbine engine component temperatures using thermographic phosphors. SPIE Newsroom.

[12] Von Moll A., Behbahani A.R., Fralick G.C., Wrbanek J.D., Hunter G.W. A Review of exhaust gas temperature sensing techniques for modern turbine engine controls. Proceedings of the 50th AIAA/ASME/SAE/ASEE Joint Propulsion Conference.

[13] Willsch M., Bosselmann T., Flohr P., Kull R., Ecke W., Latka I., Fischer D., Thiel T. Design of fiber optical high temperature sensors for gas turbine monitoring. Proceedings of the 20th International Conference on Optical Fibre Sensors.

[14] Gahan D., Fasham S., Harpin A. High temperature fiber optic pressure sensors for engine dynamics and health monitoring. Proceedings of the 2009 IEEE Avionics, Fiber-Optics and Photonics Technology Conference.

[15] Pulliam W.J., Russler P.M., Fielder R.S. 2002 High-temperature high-bandwidth fiber optic MEMS pressure-sensor technology for turbine engine component testing. Proceedings of the Environmental and Industrial Sensing.

[16] Lightweight Fiber Optic Sensors for Real-Time Strain Monitoring, NASA Technology Transfer Program; Bringing NASA Technology Down to Earth. https://technology.nasa.gov/patent/DRC-TOPS-9.

[17] Habisreuther T., Elsmann T., Graf A., Schmidt M.A. (2016). High-Temperature Strain Sensing Using Sapphire Fibers with Inscribed First-Order Bragg Gratings. IEEE Photon. J..

[18] Neumann M., Dreier F., Günther P., Wilke U., Fischer A., Büttner L., Holzinger F., Schiffer H.P., Czarske J. (2015). A laser-optical sensor system for blade vibration detection of high-speed compressors. Mech. Syst. Signal Process..

[19] Tatam R.P., Grattan K.T.V., Meggitt B.T. (1995). Optical fiber modulation techniques for single mode fiber sensors. Optical Fiber Sensor Technology.

[20] Gopal V., Annamdas M. (2011). Review on Developments in Fiber Optical Sensors and Applications. Inter. J. Mater. Eng..

[21] Rajan G. (2020). Optical Fiber Sensors: Advanced Techniques and Applications.

[22] Lumens P.G.E. (2014). Fibre-optic sensing for application in oil and gas wells. Ph.D. Thesis.

[23] Johny J., Amos S., Prabhu R. (2021). Optical Fibre-Based Sensors for Oil and Gas Applications. Sensors.

[24] Rajeev P., Kodikara J., Chiu W.K., Kuen T. (2013). Distributed Optical Fibre Sensors and Their Applications in Pipeline Monitoring. Key Eng. Mater..

[25] Cooperman A., Martinez M. (2015). Load Monitoring for Active Control of Wind Turbines. Renew. Sust. Energ. Rev..

[26] James S.W., Kissinger T., Weber S., Mullaney K., Chehura E., Pekmezci H.H., Barrington J.H., E Staines S., Charrett T.O.H., Lawson N.J. (2022). Fibre-optic measurement of strain and shape on a helicopter rotor blade during a ground run: 1. Measurement of strain. Smart Mater. Struct..

[27] Wu T., Liu G., Fu S., Xing F. (2020). Recent Progress of Fiber-Optic Sensors for the Structural Health Monitoring of Civil Infrastructure. Sensors.

[28] Gary P., Kiyoung C. (1994). Challenges to design and demonstrate fiber optic sensors on an aircraft engine. SPIE Fly-By-Light.

[29] Murugan M., Walock M., Ghoshal A., Knapp R., Caesley R. (2021). Embedded Temperature Sensor Evaluations for Turbomachinery Component Health Monitoring. Energies.

[30] Genuchten E.V., Alvarez J.M., Eesbeek S. (2017). Multi-parameter fibre optic sensing system for remote condition operation monitoring of gearbox bearings in rack and pinion jacking systems. J. Struct. Health Monit..

[31] Yates M.K. Positive displacement fuel pumping for aero-engines. Proceedings of the 11th European Fluid Machinery Congress.

[32] Chehura E., James S.W., Staines S., Groenendijk C., Cartie D., Portet S., Hugon M., Tatam R.P. (2020). Production process monitoring and post-production strain measurement on a full-size carbon-fibre composite aircraft tail cone assembly using embedded optical fibre sensors. Meas. Sci. Technol..

[33] Buggy S., James S.W., Staines S.E., Carroll R., Kitson P., Farrington D., Drewett L., Jaiswal J., Tatam R.P. (2016). Railway track component condition monitoring using optical fibre Bragg grating sensors. Meas. Sci. Technol..

[34] Lawson N.J., Correia R.N.G., James S.W., Partridge M., Staines S.E., Gautrey J.E., Garry K.P., Holt J.C., Tatam R.P. (2016). Development and application of optical fibre strain and pressure sensors for in-flight measurements. Meas. Sci. Technol..

[35] Kashyap R. (2009). Fiber Bragg Gratings.

[36] Malo B., Albert J., Hill K.O., Bilodeau F., Johnson D.C. (1994). Effective index drift from molecular hydrogen diffusion in hydrogen-loaded optical fibres and its effect on Bragg grating fabrication. Electron. Lett..

[37] Cranch G.A., Flockhart G.M.H., Kirkendall C.K. Efficient Large-Scale Multiplexing of Fiber Bragg Grating and Fiber Fabry-Pérot Sensors for Structural Health Monitoring Applications. Proceedings of the Nondestructive Evaluation for Health Monitoring and Diagnostics.

[38] Rao Y.-J. (1997). In-fibre Bragg grating sensors. Meas. Sci. Technol..

[39] Adamovsky G., Lyuksyutov S.F., Mackey J.R., Floyd B.M., Abeywickrema U., Fedin I., Rackaitis M. (2012). Peculiarities of thermo-optic coefficient under different temperature regimes in optical fibers containing fiber Bragg gratings. Opt. Comm..

[40] Spirin V.V., Shlyagin M.G., Miridonov S.V., Marquez I. (2001). Temperature-insensitive strain measurement using differential double Bragg grating technique. Opt. Laser Technol..

[41] Flockhart G.M.H., Maier R.R.J., Barton J., MacPherson W.N., Jones J.D.C., Chisholm K.E., Zhang L., Bennion I., Read I., Foote P.D. (2004). Quadratic behavior of fiber Bragg grating temperature coefficients. Appl. Opt..

[42] Metals—Temperature Expansion Coefficients. https://www.engineeringtoolbox.com.

[43] https://www.smartfibres.com/products/smartscan.

[44] https://www.3m.co.uk/3M/en_GB/p/d/b40067978/.

[45] https://www.artisantg.com/info/Dewetron_DAQP_BRIDGE_B_Manual_2019122115450.pdf.

[46] Wild G. Optical Fiber Bragg Grating Sensors Applied to Gas Turbine Engine Instrumentation and Monitoring. Proceedings of the 2013 IEEE Sensors Applications Symposium.

[47] Chehura E., James S.W., Tatam R.P. (2007). Temperature and strain discrimination using a single tilted fibre Bragg grating. Opt. Comm..

[48] Stepanov K.V., Zhirnov A.A., Sazonkin S.G., Pnev A.B., Bobrov A.N., Yagodnikov D.A. (2022). Non-Invasive Acoustic Monitoring of Gas Turbine Units by Fiber Optic Sensors. J. Sens..

[49] Nicchiotti G., Page S.A., Solinski K., Andracher L., Paulitsch N., Giuliani F. 2021 Characterisation and validation of an optical pressure sensor for combustion monitoring at low frequency. Proceedings of the ASME Turbo Expo 2021: Turbomachinery Technical Conference and Exposition.

[50] https://gtr.ukri.org/projects?ref=113095#/tabOverview.

